# An Unusual Case of Dietary-Induced Liver Injury during Pregnancy: A Case Report of Probable Liver Injury due to High-Dose Turmeric Intake and Literature Review

**DOI:** 10.1155/2024/6677960

**Published:** 2024-02-06

**Authors:** Kareem Haloub, Elly McNamara, Rani Haj Yahya

**Affiliations:** The University of Melbourne, Parkville, VIC 3052, Australia

## Abstract

Turmeric-induced liver injury is a controversial topic, and turmeric is safe to consume during pregnancy in small amounts; however, it might be an uncommon cause of liver injury if consumed in large amounts. We hereby report a case of a pregnant patient who demonstrated atypical signs and symptoms of dietary-induced liver injury during pregnancy. She presented with itching at 23 weeks 4 days of pregnancy and had deranged liver function tests and was diagnosed with dietary-induced liver injury. The patient was managed with a strict diet during the pregnancy which resulted in a significant improvement in the clinical and biochemical findings during the pregnancy.

## 1. Background

Turmeric is an herb that people have used for thousands of years for both flavour and medicinal properties. Turmeric root has anti-inflammatory effects and is rich in antioxidants. Studies on pregnant rodents, as well as in vitro studies, emphasized the numerous biological activities of curcumin [1]. Curcumin appeared to ameliorate diabetes in a GDM mouse model, as well as a rat model, and was found to be neuroprotective against environmental toxic agents [1], and researchers have not carried out studies on the safety of turmeric consumption during pregnancy in humans [2].

## 2. Case Presentation

A pregnant patient, Gravida 3, Para 1, presented at 23 weeks and 4 days of gestation with a history of one week of generalized itching all over her body. The patient had a previous uncomplicated pregnancy and denied any personal or family history of liver disease. She also denied any history of headache, blurred vision, jaundice, change in urine or stool colour, chills, rigors, nausea, vomiting, or abdominal pain. The patient did not consume alcohol during pregnancy or use medication; however, she consumed turmeric 3-4 times per day (5–10 grams) for the first time due to sore throat. Laboratory investigations revealed elevated liver enzymes: alanine aminotransferase 734 U/L, alkaline phosphatase 175 U/L, gamma-glutamyl transferase 40 U/L, and high nonfasting serum bile acid levels 73 *μ*mol/L. She was admitted to the hospital for further workup and management. Viral serologies including EBV, toxoplasmosis, herpes simplex virus, cytomegalovirus, HIV, parvovirus, and hepatitis profile (A, B, C, D, and E) were negative, blood count and coagulation profile were within normal range, preeclampsia workup was negative, and autoimmune antibodies for liver and kidney diseases were negative. Imaging modalities including abdominal ultrasound and MRCP could not identify any significant abnormality that might be related to the clinical findings (all the investigations are summarized in Table 1). Ursodeoxycholic acid 250 mg QID commenced as atypical presentation of early-onset intrahepatic cholestasis of pregnancy could not be fully excluded. However, this was unlikely due to early gestational age and significantly high transaminase levels. The patient was also asked to commence a restricted diet—Mediterranean diet, no spices/turmeric (and no any kind of food that might be related to liver injury)—as there was a presumption that the clinical presentation might be related to diet-induced liver injury. She was discharged home at 24 weeks and 6 days of gestation. The patient continued to have weekly outpatient follow-up in our maternal-fetal medicine and gastroenterology clinics to monitor her symptoms and liver function tests and for fetal growth surveillance. Over the course of the pregnancy, her symptoms resolved and her liver enzymes including bile acid levels stabilised and reached almost normal levels around 32 weeks of gestation, and this made the presumed diagnosis of diet-induced liver injury probable.

At 35 weeks of pregnancy, the patient presented with hands and palms itching, she stated she was following the medical advice regarding strict Mediterranean diet and has not been having turmeric or any other spices as advised, the liver function tests started to be deranged again, and the serum bile acids increased, which raised the probability of a new diagnosis of intrahepatic cholestasis of pregnancy which is not related to her previous medical history as shown in Table 2. Given her fasting serum bile acids were elevated to 64 *μ*mol/L despite being on ursodeoxycholic acid and restricted diet, the patient delivered vaginally at 37 weeks following an induction of labour and a 2916 grams healthy female was born.

### 2.1. Differential Diagnosis If Relevant

The differential diagnosis included early-onset intrahepatic cholestasis of pregnancy, which is still considered as an uncommon presentation, and dietary-induced liver injury with unclear etiology, the patient was on restricted diet with no herbs including turmeric, and a marked improvement in LFTs and serum bile acids was noticed.

### 2.2. Outcome and Follow-Up

The patient was monitored for 2 days after giving birth, and a marked improvement in the liver function was noticed; the plan was for a follow-up in 4 weeks in the gastroenterology department to repeat serum biochemistry and commence turmeric intake as a trial to monitor the liver function test; liver function test was repeated, normal results revealed, and all the investigations and the imagining modalities were reviewed before recommencing turmeric intake. The patient stated that she commenced turmeric in small amounts added to food but not similar amount to what she ate before (less than 1 small tablespoon), and the follow-up of liver function tests was within normal range; when she was asked to have similar amount, she declined as she was stressed and anxious it might affect her liver.

## 3. Discussion Include a Very Brief Review of Similar Published Cases

Approximately 3 percent of all pregnancies are complicated by liver function test abnormalities. A database from the USA that included over 40 million pregnancy hospitalizations from 2002 to 2010 found that the liver disease diagnoses that can affect the pregnancy can vary such as diseases bundled together as liver disorders of pregnancy (i.e., acute fatty liver of pregnancy, intrahepatic cholestasis of pregnancy, and postpartum hepatorenal syndrome, HELLP) or can be presented during the pregnancy (i.e., gallbladder disease, cholelithiasis, hepatitis C virus infection, biliary tract disease (biliary obstruction, cholangitis), hepatitis B virus infection, haemolysis, and alcohol-related liver disease); however, the prevalence data for liver disease in pregnant patients who do not require hospitalization are lacking [4]. Elevated liver biochemical and function tests in pregnant patients may pose a challenge for the clinicians as it might be related or unrelated to the pregnancy itself; thus, it requires a diagnostic evaluation similar to the evaluation of the nonpregnant patients while also taking into consideration the expected physiological changes of the pregnancy as well as pregnancy-related liver disorders such as intrahepatic cholestasis, HELLP, and acute fatty liver. During the last few decades, there has seen a rise in the use of herbal supplements, natural products, and traditional medicines; however, there are growing concerns related to the safety, toxicities, and the amount of these medicines. Clinical manifestations range from asymptomatic cases with abnormal liver function tests to a sudden and severe liver failure necessitating liver transplantation [5]. There are no precise estimates of the frequency of hepatotoxicity attributable to herbal and dietary supplements. Patients often do not report the use of herbal products to their clinicians. They self-medicate with large amounts [6], and the untoward effects of using herbs in pregnancy can vary from heartburn and allergic reaction to miscarriage, premature labour, and even theoretical concerns regarding birth defects (green tea might interfere with serum folate metabolism) [7].

The United States Drug-Induced Liver Injury Network (DILIN) found that approximately 15 to 20 percent of cases of drug-induced liver injury (DILI) could be attributed to herbal and dietary supplements [8]. Herb-induced liver injury typically presents as acute hepatocellular injury with elevated aminotransferases, bilirubin, and jaundice [9]. However, it may also present as asymptomatic liver enzyme elevations, acute or chronic hepatitis (symptomatic malaise, nausea, vomiting, abdominal pain, etc.), acute liver failure with coagulopathy and encephalopathy, or signs and symptoms of cirrhosis. The list of herbal medications and dietary supplements associated with hepatic injury is long (i.e., Pyrrolizidine alkaloids, Ayurvedic herbs, Germander Greater celandine, Chaparral, and green tea extracts), and a more complete listing can be found in the searchable database of drugs, herbal medications, and dietary supplements developed by the National Institutes of Health [10].

Turmeric is an herb that has long been recognized for its medicinal properties and has received interest from both the medical and scientific world and from culinary enthusiasts as it is the major source of polyphenol curcumin. It aids in the management of oxidative and inflammatory conditions, metabolic syndrome, arthritis, anxiety, and hyperlipidemia [3]. Current evidence suggests that curcumin is a highly pleiotropic molecule with numerous targets and mechanisms of action. It has properties that alter the activity of enzymes (the activity of COX-2 activity, lipoxygenase, and the iNOS enzyme), growth factor receptors (DR5 expression, p38-dependent upregulation, and TNF-*α*), cofactors, and other molecules [11].

Curcumin has the potential to prevent and/or manage various diseases due to its anti-inflammatory, antioxidant, and antiapoptotic properties with an excellent safety profile [12]. It has been extensively studied in various fields, showing a wide range of action which was demonstrated by in vitro studies and animal models suggesting the use of this compound as a therapeutic agent in counteracting several pregnancy complications, and for this reason, curcumin could play a key role in improving pregnancy outcome in these complications [13]. Turmeric has years of traditional use, and there are some preliminary convincing research studies, but there is no standard dosage recommended, and it is suggested that 500−2,000 mg of turmeric extract per day is generally considered safe for pregnant women [14, 15].

A wide variety of preclinical studies support the effectiveness of dietary curcumin in the management of oxidative associated liver diseases; however, there are only few RCTs assessing the efficacy of curcumin in liver disorders. Further well-designed RCTs are required to confirm the dietary and adjunctive role of curcumin as a promising protective or curative agent in the management of oxidative associated liver disease [16]. Products containing turmeric are generally listed by the curcuminoid content, commonly making up only 3–5% of turmeric rhizome and giving turmeric its yellow appearance [17]. It is widely used due to its therapeutic effectiveness and acceptable safety specification [18, 19]. On the contrary, turmeric causes potentially severe liver injury that is typically hepatocellular with a latency of 1 to 4 months either to usage or in combination with black pepper, and there is a strong linkage to HLAB^*∗*^35 : 01 [20]. Gianmarco reported that no solid evidence exists that the combination of curcumin and piperine could be the cause of hepatotoxicity. Also, for the specific case of curcumin, the number of cases reported is still too limited for definitive answer and only a more extensive clinical trial in the presence of bioavailability enhancers could definitively settle this dispute [21].

Furthermore, Niccolo in recent case series and systematic review of hepatotoxicity due to turmeric revealed an association between high bioavailability and high dosage of curcumin/curcuminoids and liver injury, and the causal relationship was also supported by the positive dechallenge observed in most cases. The evaluation of Italian cases of *Curcuma longa*-induced acute hepatitis confirmed the association between *Curcuma longa* and liver injury [22]. Few other case reports have concluded that pure turmeric might directly lead to liver injury but stated that unknown contaminants causing hepatic injury could not be excluded [23].

Lastly, the use of other different herbs in pregnancy such as garlic, sage, and ginger is also controversial. While these herbs can be safely used in pregnancy in amounts found in food, larger amount could potentially be harmful [12].

## 4. Summary

The presented case is “probable” for turmeric-induced liver injury. The timing with respect to onset and offset of liver injury was compatible; the patient underwent extensive serological screening to exclude other causes of deranged liver function; all the investigations were within normal range. That is why a strict dietary modification commenced and showed significant improvement in the liver injury.

## 5. Learning Points/Take Home Messages

- Liver injury in pregnant patients may pose a challenge for clinicians as it includes a wide range of differential diagnosis
- Curcumin/turmeric causes potentially severe liver injury in high doses
- Further high-quality studies are needed to establish the safety of using herbs and their effectiveness during pregnancy and the amount which can be considered safe

## Figures and Tables

**Table 1:** Laboratory and imaging results.

Investigations	Results	Comments	Normal levels
Hemoglobin	120 g/l	Within normal range	98–137 g/L
Platelets	209 × 10*e*^9^/L	Within normal range	150–400 × 10*e*^9^/L
White cell count	10.4 × 10*e*^9^/L	Within normal range	5.9–16.9 × 10*e*^9^/L
Serum sodium	137 mmol/L	Within normal range	133–139 mmol/L
Serum potassium	4.0 mmol/L	Within normal range	3.7–4.7 mmol/L
Urea	2.8 mmol/L	Within normal range	0.9–4.5 mmol/L
Creatinine	34 *μ*mol/L	Within normal range	20–90 *μ*mol/L
Alanine aminotransferase	734 U/L	Abnormal	<35 U/L
Alkaline phosphatase	175 U/L	Abnormal	35–127 U/L
Gamma-glutamyl transferase	40 U/L	Within normal range	8–78 U/L
Total bilirubin levels	4 *μ*mol/L	Within normal range	0–15 *μ*mol/L
Unconjugated bilirubin	4 *μ*mol/L	Within normal range	0–10 *μ*mol/L
Cytomegalovirus Ab.IgM	Not detected		
EBV VCA IgM antibodies	Not detected		
Hepatitis profile (A, B, C, D, E)	Negative		
HSV IgM total antibodies (EIA)	Not detected		
Coagulation profile	Within normal range		
ANA screen	Negative		
Smooth muscle Ab	Negative		
Mitochondrial Ab	Negative		
Liver kidney microsome Ab	Negative		
Extractable nuclear Ab screen	Negative		
HIV serology	Negative		
dsDNA Ab (Farr) interpretation	Negative		
Urine analysis	Unremarkable		
Urine protein/creatinine ratio	11.7	Negative	<30 mg/mmol
Gliadin peptide (DGP) IgG	1.2 U/ml	Negative	0–20 U/mL

Abdominal ultrasound	Unremarkable	Incidental 16 mm echogenic lesion within segment VIII has sonographic appearances of a probable benign haemangioma. No cause found for patient's raised LFTs	
		Gallbladder contains mobile debris, no sonographic evidence of cholelithiasis or cholecystitis	

MRI cholangiogram		No biliary dilatation or choledocholithiasis. Cystic pancreatic lesions are compatible	
		With side-branch IPMN, with no worrisome features on this study	

Obstetric ultrasound		Normal growth and wellbeing	
		EFW is on the 29^th^ percentile with normal fetal movements, AFI and Doppler	

**Table 2:** Laboratory results.

Investigations	Results	Comments	Normal levels
Hemoglobin	120 g/l	Within normal range	98–137 g/L
Platelets	209 × 10*e*^9^/L	Within normal range	150–400 × 10*e*^9^/L
White cell count	10.4 × 10*e*^9^/L	Within normal range	5.9–16.9 × 10*e*^9^/L
Serum sodium	133 mmol/L	Within normal range	133–139 mmol/L
Serum potassium	4.3 mmol/L	Within normal range	3.7–4.7 mmol/L
Urea	3.0 mmol/L	Within normal range	0.9–4.5 mmol/L
Creatinine	36 *μ*mol/L	Within normal range	20–90 *μ*mol/L
Alanine aminotransferase	51 U/L	Abnormal	<35 U/L
Alkaline phosphatase	266 U/L	Normal	35–262 U/L
Gamma-glutamyl transferase	25 U/L	Within normal range	8–78 U/L
Total bilirubin levels	3 *μ*mol/L	Within normal range	0–15 *μ*mol/L
Unconjugated bilirubin	3 *μ*mol/L	Within normal range	0–10 *μ*mol/L
Prothrombin time	12.4	Within normal range	11.5–14.5 seconds
INR	0.9	Within normal range	0.8–1.2 ratio
APTT	31	Within normal range	27–39 seconds
Fibrinogen	6.5 g/l	Within normal range	3.0–8.0 g/l

## Data Availability

No data were used to support this study.
